# Heterogeneity estimation in meta-analysis of standardized mean differences when the distribution of random effects departs from normal: A Monte Carlo simulation study

**DOI:** 10.1186/s12874-022-01809-0

**Published:** 2023-01-17

**Authors:** Desirée Blázquez-Rincón, Julio Sánchez-Meca, Juan Botella, Manuel Suero

**Affiliations:** 1 Department of Basic Psychology and Methodology, Faculty of Psychology, University of Murcia, Murcia, Spain; 2 Department of Social Psychology and Methodology, Faculty of Psychology, Autonomous University of Madrid, Madrid, Spain

**Keywords:** Heterogeneity, Non-normality, Random effects, Meta-analysis, Between-study variance, Simulation study

## Abstract

**Background:**

Advantages of meta-analysis depend on the assumptions underlying the statistical procedures used being met. One of the main assumptions that is usually taken for granted is the normality underlying the population of true effects in a random-effects model, even though the available evidence suggests that this assumption is often not met. This paper examines how 21 frequentist and 24 Bayesian methods, including several novel procedures, for computing a point estimate of the heterogeneity parameter (${\tau }^{2}$) perform when the distribution of random effects departs from normality compared to normal scenarios in meta-analysis of standardized mean differences.

**Methods:**

A Monte Carlo simulation was carried out using the R software, generating data for meta-analyses using the standardized mean difference. The simulation factors were the number and average sample size of primary studies, the amount of heterogeneity, as well as the shape of the random-effects distribution. The point estimators were compared in terms of absolute bias and variance, although results regarding mean squared error were also discussed.

**Results:**

Although not all the estimators were affected to the same extent, there was a general tendency to obtain lower and more variable ${\tau }^{2}$ estimates as the random-effects distribution departed from normality. However, the estimators ranking in terms of their absolute bias and variance did not change: Those estimators that obtained lower bias also showed greater variance. Finally, a large number and sample size of primary studies acted as a bias-protective factor against a lack of normality for several procedures, whereas only a high number of studies was a variance-protective factor for most of the estimators analyzed.

**Conclusions:**

Although the estimation and inference of the combined effect have proven to be sufficiently robust, our work highlights the role that the deviation from normality may be playing in the meta-analytic conclusions from the simulation results and the numerical examples included in this work. With the aim to exercise caution in the interpretation of the results obtained from random-effects models, the *tau2()* R function is made available for obtaining the range of ${\tau }^{2}$ values computed from the 45 estimators analyzed in this work, as well as to assess how the pooled effect, its confidence and prediction intervals vary according to the estimator chosen.

**Supplementary Information:**

The online version contains supplementary material available at 10.1186/s12874-022-01809-0.

## Background

Meta-analysis is the set of procedures aimed at synthesizing the combined evidence from multiple scientific studies that attempt to answer a common research question. Combining the evidence, rather than relying on individual studies, has important benefits like an increase in the statistical power to detect an effect of interest or the possibility to examine why and how individual estimates vary. However, these benefits only apply when the assumptions underlying the statistical procedures used in meta-analysis are met or, at least, when these procedures are robust enough to the violation of these assumptions. One of the main assumptions that is usually taken for granted in applied meta-analysis is the normality underlying the population of true effect sizes in a random-effects model. Previous works have tried to answer how the estimation and inference regarding the pooled effect size perform under non-normal random effects [1–3], but less has been said about other important parameters, like the heterogeneity or between-study variance.

This paper presents a Monte Carlo simulation that examines how the available methods for computing a point estimate of the between-study variance perform when the distribution of effect sizes departs from normality in meta-analyses of standardized mean differences.

### The role of the heterogeneity parameter

The random-effects model assumes that the effect estimates collected in the meta-analysis may not only vary due to random sampling error (given the primary studies employ samples of different size), but also because each study is estimating a different parametric effect. Continuing with its mathematical formulation, the effect size estimate ${\widehat{\theta }}_{i}$ of the ${i}^{th}$ study ($i=1,\dots ,k$) is decomposed into ${\theta }_{i}+{e}_{i}$, where ${\theta }_{i}$ refers to the parametric effect estimated, and ${e}_{i}$ represents the error or difference between ${\theta }_{i}$ and ${\widehat{\theta }}_{i}$. The expected value of the ${e}_{i}$ errors is assumed to be zero (the effect size estimators are usually unbiased) and their variance is called error variance (i.e., within-study variance, ${\sigma }_{i}^{2}$). The within-study variability is a way of quantifying the imprecision (variability due to random sampling error) of a study through a function of the study’s sample size. At the same time, the parametric effect ${\theta }_{i}$ is decomposed into ${\mu }_{\theta }+{u}_{i}$, where ${\mu }_{\theta }$ refers to the mean of the parametric effect distribution, and ${u}_{i}$ represents the difference between ${\theta }_{i}$ and ${\mu }_{\theta }$. Again, the expected value of the ${u}_{i}$ errors is assumed to be zero and their variance is called heterogeneity (i.e., between-study variance, ${\tau }^{2}$). Therefore, the between-study variance is the variability found among effect estimates that is not due to random sampling error, but to the variability present in the parametric effect (hereinafter also referred to as random-effects or true effects) distribution.

As Higgins et al. [4] (p139) stated, ‘The naive presentation of inference only on the mean of the random-effects distribution is highly misleading. Estimation of [heterogeneity] is just as important’. It could be argued that the heterogeneity is equally important as the pooled effect size, given it is needed for understanding the consistency (i.e., the homogeneity or similarity) among the effects sizes and, also, for comparing the different sources of variability underlying the distribution of effect estimates. The correct estimation of the heterogeneity parameter is decisive, not only because the pooled effect, its confidence and prediction intervals, and indices such as ${I}^{2}$ depend on it, but also because it allows us to know whether additional analyses (i.e., meta-regression, location-scale, or network meta-analyses) are needed to investigate the sources of this heterogeneity [5–7].

### The normality assumption in the random-effects model and the point estimation of ${{\varvec{\tau}}}^{2}$

As explained above, the random-effects model implies that the parameters that describe the distribution of the effect estimates are ${\mu }_{\theta }$, ${\tau }^{2}$ and ${\sigma }_{i}^{2}$, but no distributional assumption has been made to this point, neither for ${\widehat{\theta }}_{i}$ nor for ${\theta }_{i}$. The first normality assumptions are made at the within-study level, when assuming ${\widehat{\theta }}_{i}\sim N({\theta }_{i},{\sigma }_{i}^{2})$, which is often done when the primary studies have a sufficiently large sample size or when ${e}_{i}$ are supposed to be normally distributed with mean equal to 0 and variance ${\sigma }_{i}^{2}$ [8]. Since our work is focused on the normality assumption at the random-effects distribution or between-study level and not at the fixed-effect or within-study level, we refer the reader to the work of Jackson and White [8] for a comprehensive analysis of the statistical reasons why researchers often assume normality at the within-study level and the extent to which these assumptions may affect the meta-analytic results. At the between-study level, the normality assumption regarding the modelling of the random effects is made depending on the heterogeneity estimator chosen. As we will see below, there are lots of ways for estimating the between-study variance [9], and some of these estimators assume that the errors ${e}_{i}$ and ${u}_{i}$ are normally distributed and, consequently, that ${\widehat{\theta }}_{i}$ and ${\theta }_{i}$ are also normally distributed, ${\widehat{\theta }}_{i}\sim N({\theta }_{i},{\sigma }_{i}^{2})$ and ${\theta }_{i}\sim N({\mu }_{\theta },{\tau }^{2})$.

Since the generalization of random-effects models to the field of meta-analysis, new and old procedures have been used to estimate ${\tau }^{2}$. The procedures discussed in this paper are presented in Table 1, along with their abbreviation, the authors who initially developed them and the year of publication [10–29]. As can be seen in Table 1, these procedures differ according to whether they are obtained iteratively or analytically, whether they produce only positive estimates or also include zero, whether they rely on random-effects normality, and the estimation method they are based on. Since describing each procedure in detail is beyond the scope of this paper, readers are referred to the supplementary material of the study of Boedeker & Henson [30] and the work of Zhang et al., [31] where most of the estimators evaluated in the present work are described. With respect to the most novel procedures (i.e., MPM and GENQM), the logic behind its calculation is presented in the work of Viechtbauer (2021) [19]. It is also worth mentioning that the tables and figures of the present work are available in the supplementary material hosted in the Open Science Framework repository [32].

**Table 1:** Point estimators for the heterogeneity parameter

***Point estimator for***${{\varvec{\tau}}}^{2}$		***Author (year)***	***Computation***	***Range***	***Assume*** ***normality***	***Estimation method***
Cochran (Hedges-Olkin)	CA	Cochran (1954) [10]	Direct	Non-negative	No	Method of the moments
Mandel-Paule	MP	Mandel & Paule (1970/82) [11, 12]	Iterative	Non-negative	No	Method of the moments
DerSimonian-Laird	DL	DerSimonian & Laird (1986) [13]	Direct	Non-negative	No	Method of the moments
Hartung-Makambi	HM	Hartung & Makambi (2002) [14]	Direct	Positive	No	Method of the moments
Two-step Cochran	CA2	DerSimonian & Kacker (2007) [15]	Direct	Non-negative	No	Method of the moments
Two-step DerSimonian-Laird	DL2	DerSimonian & Kacker (2007) [15]	Direct	Non-negative	No	Method of the moments
Positive DerSimonian-Laird	DLp	Kontopantelis et al. (2013) [16]	Direct	Positive	No	Method of the moments
Lin-Chu-Hodges *r*	LCHr	Lin et al. (2017) [17]	Iterative	Non-negative	No	Method of the moments
Lin-Chu-Hodges *m*	LCHm	Lin et al. (2017) [17]	Iterative	Non-negative	No	Method of the moments
Multistep DerSimonian-Laird	DLm	vanAert & Jackson (2018) [18]	Direct	Non-negative	No	Method of the moments
Median-unbiased Mandel-Paule	MPM	Viechtbauer (2021) [19]	Iterative	Non-negative	No	Method of the moments
Median-unbiased Gen. *Q*	GENQM	Viechtbauer (2021) [19]	Iterative	Non-negative	No	Method of the moments
Maximum likelihood	ML	Hardy & Thompson (1996) [20]	Iterative	Non-negative	Yes	Maximum likelihood
Restricted maximum likelihood	REML	Viechtbauer (2005) [21]	Iterative	Non-negative	Yes	Maximum likelihood
Sidik-Jonkman	SJ	Sidik & Jonkman (2005) [22]	Direct	Non-negative	Yes	Least squares
Sidik-Jonkman (prior CA estimation)	SJ(CA)	Sidik & Jonkman (2007) [23]	Direct	Positive	Yes	Least squares
Non-parametric bootstrap DerSimonian-Laird	DLb	Kontopantelis et al. (2013) [16]	Direct	Non-negative	No	Non-parametric
Malzahn-Böhning-Holling	MBH	Malzahn et al. (2000) [24]	Direct	Non-negative	No	Non-parametric
Hunter-Schmidt (weighted by inversed variance)	HSiv	Hunter & Schmidt (1990) [25]	Direct	Non-negative	No	Artifact correction
Hunter-Schmidt (weighted by sample size)	HSss	Hunter & Schmidt (1990) [25]	Direct	Non-negative	No	Artifact correction
Hunter-Schmidt (corrected by small sample size)	HSk	Morris et al. (2015) [33]	Direct	Non-negative	No	Artifact correction
Fully Bayesian	FB	Smith et al. (1995) [26]	Iterative	Non-negative	Yes	Bayesian
Rukhin Bayes	RB	Rukhin (2013) [27]	Direct	Non-negative	No	Bayesian
Rukhin Bayes positive	RBp	Rukhin (2013) [27]	Direct	Positive	No	Bayesian
Bayes Modal	BM	Chung et al. (2013a, 2013b) [28, 29]	Iterative	Positive	Yes	Bayesian

Heterogeneity point estimators included in the present study, their abbreviation, authors and year of publication, type of calculation required to obtain the corresponding estimate, the range of real values for the${\tau }^{2}$estimates obtained, whether they assume or not normality assumptions regarding the random-effects distribution, and the underlying estimation method they are based on

### Frequentist heterogeneity estimators

Beginning with the frequentist estimators based on the method of moments, Kacker [34] showed that several methods for estimating the between-study variance were special cases of the application of the method of moments for a generalized version of the $Q$ statistic proposed by Cochran [10]:

1 $$Q_G\;=\;{\textstyle\sum_{i=1}^k}a_{1}\left({\widehat\theta}_i-{\widehat\theta}_w\right)^2,$$

where ${\widehat{\theta }}_{i}$ and ${a}_{i}$ represents the effect estimate and the weighting factor (any positive constant), respectively, for the ${i}^{th}$ study, and ${\widehat{\theta }}_{w}$ is computed as the weighted mean of the effect size estimates, ${\sum }_{i=1}^{k}{a}_{i}{\widehat{\theta }}_{i} /{\sum }_{i=1}^{k}{a}_{i}$.

Those estimators based on the generalized $Q$ statistic (CA, MP, DL, HM) are consequently obtained by setting different weights to ${a}_{i}$, and clearing the between-study variance component from the expected value of the resulting $Q$ statistic. Several other estimators (CA2, DL2, DLp, DLm) are extensions of the original procedures mentioned above, in the sense that they are computed based on one or multiple previous estimates of the Cochran [10] and the DerSimonian-Laird [13] procedures.

The MP estimator introduced by Mandel and Paule [11, 12] is mathematically identical to the empirical Bayes estimator (EB), which was independently proposed by Morris [35]. For this reason, in the following we will refer to both procedures when naming the MP estimator.

Lin et al. [17] proposed the $r$ and $m$ estimators that rely on alternative $Q$ statistics with the aim of obtaining ${\tau }^{2}$ estimates more robust to the presence of outliers. The difference between these alternative $Q$ statistics and the one presented in Eq. 1 is that the formers are weighted sums of absolute differences instead of squared residuals, and that their residuals are computed with respect to a measure less affected by outliers than the typical weighted mean.

More recently, two new estimators have been proposed (MPM and GENQM) [19] with the aim of drawing attention to the fact that the previous methods estimate ${\tau }^{2}$ from the expected value of the generalized $Q$ statistic, when in fact this statistic follows a ${\chi }^{2}$ distribution and therefore its distribution is skewed. For this reason, Viechtbauer[19] proposed to estimate ${\tau }^{2}$ from the median of the generalized $Q$ statistic instead of its expected value.

Overall, the generalized $Q$ statistic is simply a weighted sum of residuals and, therefore, does not imply any assumption of normality, regardless of the value of ${a}_{i}$. It is worth noting that, only when $Q$ is used as a test statistic for which a ${\chi }^{2}$ distribution is assumed, then normality is assumed at the within-study level since, theoretically, a ${\chi }^{2}$ variable is the sum of several independent standard normal squared variables. As a result, all the heterogeneity estimators based on $Q$statistics, although based on potentially unrealistic assumptions (i.e., known within-study variances and unbiased effect size estimates) [8], do not involve assuming normality for the random effects.

The estimators based on maximum likelihood (ML and REML) rely on the within-study and between-study normality assumptions [21]. These estimators assumed that each individual effect estimate is normally distributed with respect to a single parametric effect, and, at the same time, each parametric effect is supposed to be normally distributed regarding the mean of the parametric effects. Consequently, ${\tau }^{2}$ estimates based on these procedures are computed by maximizing the log-likelihood function where the individual effect estimates are assumed to follow a normal distribution with mean ${\mu }_{\theta }$ and variance ${\tau }^{2}+{\sigma }_{i}^{2}$.

Those heterogeneity estimators based on weighted least squares (SJ and SJ(CA)) also rely on the within-study and between-study normality assumptions [22, 23]. These estimators were developed in the framework of a linear regression model: $Y={\beta }_{0}1+\varepsilon$, where $Y$ is a vector with the effect size estimates, ${\beta }_{0}$ is a constant that represents ${\mu }_{\theta }$, and $\varepsilon$ is the error due to the total variance. Given that one of the underlying assumptions of linear regression models is that errors must be normally distributed, $\varepsilon$ is therefore assumed to follow a $N(0,{\tau }^{2}+{\sigma }^{2}$) distribution and, consequently, the true effects and their estimates are also supposed to be normally distributed.

As for the nonparametric estimators (DLb and MBH), none of them are based on underlying normality assumptions, neither at the within-study nor at the between-study level [16, 24]. The DLb estimator consists of bootstrapping the set of effect estimates to compute the DL estimator in each sample and, finally, calculating the mean DL estimate. Given that the DL estimator is based on the $Q$ statistic and the bootstrap procedure does not imply any distributional assumption regarding the random effects, the DLb estimator is free of the normality assumption at the within and between-study levels. Similar to the CA estimator, the logic underlying the MBH procedure is the difference between the total variance of the effect estimates and the variance due to random sampling error but cannot be expressed in terms of the generalized $Q$ statistic as well as the HS estimator. As a simple difference between two sources of variability, the MBH estimator does not imply any within nor between-study distributional assumptions. Although it is worth mentioning that it acknowledges the variability due to the fact that within-study variances are unknown [36] and was designed to be used only with the standardized mean difference.

The so-called artifact correction estimators (HSiv, HSss, and HSk) compute ${\tau }^{2}$ as the difference between the total variance of the effect estimates and the variance due to random sampling error, obtained as a weighted average of the within-study variances. Although Hunter and Schmidt [25] followed the same logic for developing the HS estimators that Cochran [10] did when proposing the CA estimator, the latter can be reduced to the generalized $Q$ statistic when the weights ${a}_{i}$ are set to $1/k$ whereas the HS versions cannot. The HS estimators can be seen as a difference between variances, but no distributional assumption is made regarding the effect sizes or their estimates.

### Bayesian heterogeneity estimators

We now describe the underlying assumptions of Bayesian estimators. Three of them (RB, RBp, and BM) are derived analytically, whereas the Fully Bayesian estimators (FB) require Markov chain Monte Carlo (MCMC) processes.

As we will see below, the FB estimators [26] need to make distributional *prior* assumptions regarding the parameters to be estimated (in this case, ${\mu }_{\theta }$ and ${\tau }^{2}$) and the underlying variables whose distribution depends on these parameters (that is, ${\theta }_{i}$ and ${\widehat{\theta }}_{i}$). It is important to note that any distributional assumption can be made regarding the true effects and their estimates. However, given that some of the previously described frequentist estimators assume normality both at the within and between-study levels, in the present study fully Bayesian estimators were also based on these assumptions.

As stated by Rukhin [27], the specification of the *priors* mean allows for the very explicit form of the approximate Bayesian estimators (RB and RBp), which makes them more flexible in terms of normality assumptions. The reason behind is that the only features of the normal distribution that are used to derive these estimators are the formula for the kurtosis of a normal variable, and the assumption that the within-study variances are independent of ${\widehat{\theta }}_{i}$ and follow a ${\chi }^{2}$ distribution.

Finally, the BM estimator proposed by Chung et al. [28, 29] also relies on the within and between-study normality assumptions. This method can also be considered a penalized maximum likelihood estimator, since the ${\tau }^{2}$ estimate obtained is the resulting value that maximizes the log-likelihood function (where the individual estimates are assumed to be normally distributed) but penalized by the parameters of the gamma and uniform *priors* set up for ${\tau }^{2}$ and ${\mu }_{\theta }$, respectively.

### Evidence of the lack of normality in meta-analysis

Although there are several reasons to doubt about the fulfilment of the within-study normality assumption [8, 37], this paper focuses on the effects of non-normality at the between-study level (i.e. when the distribution of random effects deviates from normality).

Contrary to the opinion of many meta-analysts, normality for the random effects cannot be justified using the central limit theorem even when the number of studies is large [4, 38]. Indeed, Rubio-Aparicio et al. [39] reviewed 54 meta-analyses regarding the effectiveness of psychological treatments, that used effect sizes from the family of mean differences, and found that the distribution of effect estimates deviated from a normal distribution in a significant proportion of the meta-analyses analyzed. More specifically, in that review the skewness distribution of the 54 meta‐analyses presented a median value of 0.52, with 25^th^ and 75^th^ percentiles of 0.18 and 1.1 and minimum and maximum values of − 2 and 3.67, respectively. When pairing the skewness and kurtosis values for the effect estimates distribution of each study, these authors found a U-shaped relationship between skewness and kurtosis.

As a case study, we now analyze two of the meta-analyses reviewed by Rubio-Aparicio et al. [39] The meta-analyses conducted by Richards and Richardson [40] and Shadish and Baldwin [41] had a similar number of independent estimates: 33 and 30, respectively. However, while the former showed a relatively normal distribution of effect estimates (skewness = -0.01, kurtosis = -0.88, *p*-value for the Shapiro-Wilks test equaled 0.855), the latter summarized estimates whose distribution was far from normal (skewness = 2.09, kurtosis = 3.64, *p*-value for the Shapiro-Wilks test equaled $1.233\bullet {10}^{-6}$).

When the frequentist and Bayesian heterogeneity estimators described previously are computed, a similar mean estimate of ${\tau }^{2}$ is obtained for both cases (0.15 for the study of Richards and Richardson [40] and 0.18 for the study of Shadish and Baldwin [41]), while the variance of the ${\tau }^{2}$ estimates is almost forty-four times larger for latter (0.07) than for the former (0.002). In other words, estimates of ${\tau }^{2}$ range from 0.11 to 0.39 in study of Richards and Richardson, while for Shadish and Baldwin’s study ${\tau }^{2}$ estimates range from 0.002 to 1.18. A pertinent conclusion would be that the deviation from normality could be affecting the variability of the heterogeneity estimates but, is this correct? Does deviation from normality affect all estimators equally? Are the most robust estimators those that make no normality assumptions? In order to answer these questions, simulation studies are needed.

### Previous literature

Even though several simulation studies have assessed the influence of the lack of normality of the random effects on the meta-analytic results [1–3], one of the few studies that in the context of meta-analysis of standardized mean differences has reported results referring to how this lack of normality affects the estimation process of the heterogeneity parameter has been the study by Kromrey and Hogarty [42], and can therefore be considered as a precursor to the present work. These authors compared the performance of three estimators of ${\tau }^{2}$ (CA, DL and ML) and found that all of them demonstrated extreme sensitivity to violations of the assumptions of normality. Their simulation results showed that the CA estimator remained essentially unbiased under normal scenarios, whereas the ML and DL estimators evidenced substantial bias under conditions of a small number of primary studies or small sample sizes. However, under non-normal conditions the CA estimator showed the greatest bias of the three estimators. With respect to the bias of the CA estimator, these authors imply that, while under normal conditions CA was practically unbiased (maximum bias of 0.07), under non-normal conditions its performance depended on the sample size of the primary studies: *“with small samples, substantial positive bias was evident as *${\tau }^{2}$* increased, but with large samples, relatively unbiased results were obtained”.* More specifically, for conditions simulated with skewness = 2 and kurtosis = 6, for example, the estimated bias reached as high as 0.69 with ${\tau }^{2}=1$ and an average sample size of 10 but did not exceed 0.03 with samples of 200. Therefore, could the sample size of primary studies act as a protective factor for the bias of heterogeneity estimators in non-normal scenarios? With respect to the variability of ${\tau }^{2}$ estimates, Kromrey and Hogarty [42] only reported results under normal conditions and concluded that ML obtained the lowest standard errors, followed by DL, while CA showed the largest sampling errors.

Although there are no other simulation studies assessing the performance of heterogeneity estimators in non-normal parametric scenarios, there are several simulation studies [21, 30, 43–45] comparing them under normal conditions that have not always reached the same conclusions regarding which estimator has the best properties, possibly due to differences in the simulation design. Table 2 shows the values for the overall effect, the amount of heterogeneity, the number and average sample size of the primary studies included in the meta-analyses of standardized mean differences generated in these previous simulation studies, along with the number of replications per simulation condition and the heterogeneity estimators analyzed in each case. Viechtbauer [21] found that there was an inverse relationship between bias and efficiency of the estimators analysed in his simulation study and concluded that the CA procedure was unbiased across all the simulated conditions but was the one that showed the greatest mean square error (*MSE*) among the DL, HSiv, ML, and REML estimators. Novianti et al. [43] explained that CA, DL, DL2, SJ(CA), MP and REML were comparable, showing relatively small bias for small amounts of heterogeneity. But, in contrast, the SJ estimator largely overestimated the real value of ${\tau }^{2}$ in most cases. Petropoulou and Mavridis [44] compared in terms of bias twenty frequentist and Bayesian estimators. In their simulation, the DLb and DLp estimators showed to be less biased in all conditions, followed by the REML and HM. Langan et al. [45] compared estimators similar to those previous simulation studies in terms of relative bias and *MSE*. These authors found that DL2 and REML, despite having a negative bias in a small number of scenarios, performed similarly and had relatively low bias and low *MSE*compared to the other estimators, even when there were substantial differences in the sample sizes of the primary studies within the same meta-analysis. Finally, with respect to the simulation study carried out by Boedeker & Henson [30], the estimator that showed the best performance with regard to bias and *MSE* over most conditions was that of MP followed by CA, REML, RB and those fully Bayesian focused on the posterior median with *prior* $\Gamma (0.001, 0.001)$.

**Table 2:** Parameters or factors varied in previous simulation studies

Study	Heterogeneity estimators	Overall effect (${\mu }_{\theta }$)	Amount of heterogeneity (${\tau }^{2}$)	Number of studies ($k$)	Average sample size ($N$)	Replicat
Viechtbauer [21]	CA, DL, HSiv, ML, REML	0, 0.2, 0.5, 0.8	0, 0.01, 0.025, 0.05, 0.1	5, 10, 20, 40, 80	20, 40, 80, 160, 320	100,000
Novianti et al. [43]	CA, MP, DL, REML, SJ, SJ(CA), CA2, DL2	0, 0.5	From 0 to 0.0366	10, 15, 20, 30, 50	From 40 to 400	10,000
Petropoulou and Mavridis [44]	CA, MP, DL, HSiv, ML, AREML, REML, SJ, SJ(CA), CA2, DL2, HM, MBH, DLp, DLb, BM, RB, RBp, FB_informative_, FB_vague_	0, 0.3, 0.5, 0.8	0, 0.01, 0.05, 0.5	10, 20, 30, 50	From 40 to 400	1,000
Langan et al. [45]	CA, MP, DL, CA2, DL2, HM, SJ, SJ(CA), REML	0.5	From 0 to 2.44	2, 3, 5, 10, 20, 30, 50, 100	40, 220, 400, 1520, 3000	5,000
Boedeker and Henson [30]	CA, CA2, DL, DLp, DL2, HM, HSiv, SJ, MBH, PM, ML, and REML, FB_mean_, FB_median_, FB_mode_, RB, RBp, BM	0.5	0, 0.01, 0.025, 0.05, 0.075, 0.1, 0.25, 0.5, 1	5, 10, 20, 40, 60, 100	40, 80, 120	1,000

Simulation factors included in previous simulation studies assessing the performance of different heterogeneity estimators under normal random-effects conditions. Within the fully Bayesian framework, FB_*informative*_ corresponds to the *prior* specification ${\tau }^{2}\sim logN(-2.56, {1.74}^{2})$; FB_*vague*_ corresponds to the *prior* specification $\mathrm{log}({\tau }^{2})\sim t(-3.44, {2.59}^{2}, 5)$; and FB_*mode*_, FB_*mean*_, FB_*median*_ correspond to the fully Bayesian procedures also evaluated in the present simulation study to model a function of heterogeneity (five different priors for $\tau$, and other two for $1/{\tau }^{2}$) centered on the posterior mean, median and mode, respectively

These simulation studies often end up advising the use of one estimator or another depending on very specific conditions of the number of studies, the sample size, the unknown amount of heterogeneity, among other factors, although among the estimators most recommended for continuous data, as the standardized mean differences, we can find the CA, MP, and REML procedures.

In addition to all the simulation factors taken into account in the previous simulations, we consider it necessary to examine how deviation from normality affects the estimation of the heterogeneity parameter, since it is a condition evidenced in real meta-analyses. The estimation of the parameter usually involves a greater negative bias the greater the amount of real heterogeneity [21, 30, 43–45]. This deserves to be taken into account when reporting the conclusions of a meta-analysis, where often the small number of studies and sample sizes do not allow a proper estimation of ${\tau }^{2}$. Therefore, biased and very unstable ${\tau }^{2}$ estimates may be found. If the role that the deviation from random-effects normality can play is also overlooked, the conclusions of the meta-analysis can be seriously compromised.

## Methods

### Aim of the present study

In this study, we aimed to compare the performance of the heterogeneity estimators previously presented when the assumption of normality for the distribution of parametric effects is altered for several reasons.

Primarily, we would like to extend the results of Kromrey and Hogarty [42] to a larger number of estimators, especially those more novel procedures. The estimators proposed by Lin et al. [17] have only been evaluated in meta-analyses of rare binary events [46], but to our knowledge their performance remains unknown when the effect size belongs to the family of mean differences. At the same time, the estimators proposed by Viechtbauer [19] were recently developed as improved versions of previously proposed estimators but have not yet been examined or compared with other procedures.

Secondly, we would like to know how the deviation from normality influences the heterogeneity estimation. Specifically, we were interested in whether all the estimators were affected the same way and whether the estimators with better properties under normality are also the preferred ones when that assumption does not hold. Regarding the latter point, the simulation works mentioned above should help us to rank the estimators already studied in terms of their bias and efficiency in normal scenarios. However, since the results vary from one study to another, we believe that the most correct approach is to simulate new normal random-effects conditions to serve as a starting point to compare the performance of the heterogeneity estimators under non-normal conditions.

To do so, we carried out a Monte Carlo simulation study in R software [47] where we simulated data using the standardized mean difference as the outcome measure, given it is a popular index in psychology and other social and health sciences [48]. Throughout this section the data generation, the simulation factors and the outcome variables evaluated are explained and justified.

### Data generating process

Data for the primary studies were generated following a two-group (experimental and control) design with respect to a continuous dependent variable, and the outcome measure used was the standardized mean difference, also known as Cohen’s *d*. So far, the nomenclature used to refer to the parametric and estimated effect sizes has been ${\theta }_{i}$ and ${\widehat{\theta }}_{i}$, involving any effect size index. Since from now on we will only talk about the standardized mean difference, we will refer to the parametric and estimated effects as ${\delta }_{i}$ and ${g}_{i}$, respectively.

To simulate a single meta-analysis, a parametric effect size ${\delta }_{i}$ was randomly selected out of a distribution of parametric effect sizes with mean ${\mu }_{\delta }=$ 0.5 and a specific variance ${\tau }^{2}$ for each of the $k$ primary studies. In the present work, the value of ${\mu }_{\delta }$ has not been varied (included as a factor in the simulation) since previous work [21, 43, 44] has shown that it has no effect on the estimation of ${\tau }^{2}$. Next, following the work of Hedges [49], an observed Cohen’s *d* value for each primary study ${g}_{i}$ was randomly sampled from a distribution that was $1/\sqrt{{\widetilde{n}}_{i}}$ times a noncentral ${t}_{i}$ random variable, where ${\widetilde{n}}_{i}={n}_{i}^{E}\bullet {n}_{i}^{C}/({n}_{i}^{E} + {n}_{i}^{C})$, and ${n}_{i}^{E}$ and ${n}_{i}^{C}$ being the sample sizes for the experimental and control groups, respectively. The noncentral ${t}_{i}$ distribution had a noncentrality parameter $\sqrt{{\widetilde{n}}_{i}}{\delta }_{i}$ and ${m}_{i}$ degrees of freedom, where ${m}_{i}={n}_{i}^{E} + {n}_{i}^{C}-2$. Hedges and Olkin [50] showed that $g$ is a positively biased estimator of Cohen’s *d*, and proposed a nearly unbiased estimator which is computed as ${g}_{i}^{U}=c({m}_{i})\bullet {g}_{i}$, where $c({m}_{i})$ is a correction factor for small sample sizes given by.

2 $$c\left(m_i\right)=\frac{\Gamma\left(m_i/2\right)}{\sqrt{m_i/2}\bullet\Gamma\left[(m_i-1)/2\right]}.$$

Once a Cohen’s *d* unbiased estimate ${g}_{i}^{U}$ was obtained, the estimate of the within-study variance of each primary study, which corresponds to the sampling variance of ${g}_{i}^{U}$, was computed as.

3 $$\widehat\sigma_i^2\left(g_i^U\right)\;=\;\left[1/\widetilde{n}{_i}\right]\;+\;\left[1-1/\left(\frac{c\left(m_i\right)^2\bullet m_i}{m_i-2}\right)\right]\;\left(g_i^U\right)^2,$$

since the estimator presented in Eq. 3 has shown to be the least biased (although not the most efficient) of the main known estimators for the sampling variance of ${g}_{i}^{U}$ [51]. For a detailed analysis of this and other alternative estimators of the sampling variance of ${g}_{i}^{U}$ and the software tools where they are implemented, we refer readers to the work of Suero et al. [51]

### Simulation conditions

The factors manipulated in this simulation were, on the one hand, those already studied previously: the number and sample size of the primary studies, and the amount of heterogeneity. And, on the other hand, the shape of the true effects distribution, which has always been established as a normal distribution in preceding simulation works. To identify a range of realistic scenarios in the field of social and health sciences, the manipulated conditions in the current study were set according to the results of a systematic review of 54 meta‐analyses on the efficacy of psychological interventions using different types of standardized mean differences [39].

For the number of studies *k*, five values were considered, 10, 30, 50, 70, and 90, corresponding to a small‐to‐large number of studies for the meta‐analyses. To model the sample sizes of the primary studies $N$, we followed the sample size distributions of those 54 meta-analyses included by Rubio-Aparicio et al. [39] These distributions were positively skewed, with an average skewness coefficient of 1.423. To emulate a similar distribution, we used a ${\chi }^{2}$ distribution. Since the skewness coefficient for the ${\chi }^{2}$ distribution is calculated as $\sqrt{8/v}$ ($v$ representing its degrees of freedom), equaling the skewness coefficient to 1.423 results in a ${\chi }^{2}$ distribution with 3.95 degrees of freedom. In the present simulation, the average total sample size of the primary studies *N* was set to 20, 40, 60, 80, and 100. As the expected value of the ${\chi }^{2}$ equals its degrees of freedom, to model the distribution of each average sample size condition, $N-$ 3.95 was added to the values of the previous ${\chi }^{2}$ distribution, resulting in distributions with 3.95 degrees of freedom and mean $N$. From these distributions, a value for the total sample size of each primary study was randomly generated. Half of this value, rounded to the nearest integer, was the sample size for the experimental ${n}_{i}^{E}$ and control ${n}_{i}^{C}$ groups of the ${i}^{th}$ study and, therefore, ${n}_{i}^{E}={n}_{i}^{C}$.

Furthermore, a wide range of values for the population between‐studies variance or heterogeneity was considered. As found in previous reviews, estimates for ${\tau }^{2}$ in meta-analyses of health and social sciences range from 0 to 1 (or even greater values), with a higher concentration in the 0 to 0.1 range. Therefore, in the present work the values 0.000, 0.010, 0.025, 0.050, 0.075, 0.100, 0.250, 0.500, 0.750, 1.000 were considered for ${\tau }^{2}$.

**Fig. 1:**
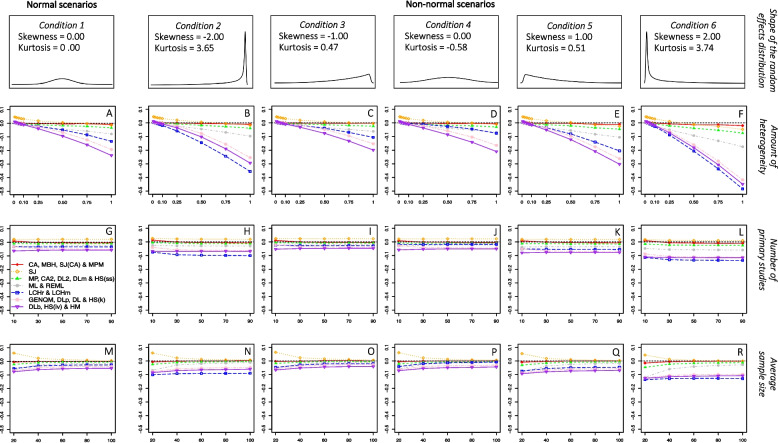
*Absolute bias of the frequentist estimators* *Note*. Absolute bias of the frequentist estimators as a function of the amount of heterogeneity, the number of primary studies, and the average sample size. The results are presented separately for each condition of the shape of the random-effects distribution. CA = Cochran estimator; MBH = Malzahn-Böhning-Holling estimator; SJ(CA) = Sidik-Jonkman estimator with prior CA estimation; MPM = median-unbiased Mandel-Paule estimator; SJ = Sidik-Jonkman estimator; MP = Mandel-Paule estimator; CA2 = two-step Cochran estimator; DL2 = two-step DerSimonian-Laird estimator; DLm = multistep DerSimonian-Laird estimator; HS(ss) = Hunter-Schmidt estimator weighted by sample size; ML = maximum likelihood estimator; REML = restricted maximum likelihood estimator; LCHr = Lin-Chu-Hodges *r* estimator; LCHm = Lin-Chu-Hodges *m* estimator; GENQM = median-unbiased generalized *Q* statistic estimator; DLp = positive DerSimonian-Laird estimator; DL = DerSimonian-Laird estimator; HS(k) = Hunter-Schmidt estimator corrected by small sample size; DLb = nonparametric bootstrap DerSimonian-Laird estimator; HS(iv) = Hunter-Schmidt estimator weighted by inversed variance; HM = Hartung-Makambi estimator

The shape of the distribution of the parametric effect sizes $s$ was manipulated through six combinations of skewness and kurtosis values. First, a normal scenario was set, where skewness and kurtosis equaled zero. Second, five nonnormal conditions were considered based on the results from Rubio-Aparicio et al. [39] The skewness distribution of the 54 meta‐analyses presented a median value of 0.52, with 25th and 75th percentiles of 0.18 and 1.1 and minimum and maximum values of − 2 and 3.67, respectively. Based on these results, a wide range of skewness values of − 2, − 1, 0, 1, and 2 were selected to simulate the parametric effect distribution. The nonlinear relationship exhibited by the 54 pairs of skewness and kurtosis values found in the systematic review was used to predict the kurtosis values. A nonlinear predictive model previously fitted to this dataset [3], lead to the predictive equation $Kurtosis = -0.581 + 0.023 * Skewness + 1.069 * {Skewness}^{2}$, resulting in five combinations of skewness and kurtosis values (− 2, 3.65), (− 1, 0.47), (0, − 0.58), (1, 0.51), and (2, 3.74). We used the *rpearson()* function from the *PearsonDS* package [52] to generate random values from a distribution of parametric effect sizes with mean 0.5, and a given variance, skewness, and kurtosis, which is based on the Pearson distribution system [53]. Fig. 1 presents the probability density functions of the parametric effect size distributions for the six simulated combinations of skewness and kurtosis, with ${\mu }_{\delta }=0.5$ and ${\tau }^{2}=0.05$.

Table 3 summarizes the parameters being varied in this simulation work. In the end, the total number of conditions was 1350 [5 ($k$ values) × 5 ($N$) × 9 (${\tau }^{2}$) × 6 ($s$)], and for each one 1,000 meta‐analyses were generated. Therefore, 1,350,000 meta‐analyses were simulated.

**Table 3:** Parameters or factors varied in the present simulation work

***Factor***		***Values***
Overall effect	(${\mu }_{\theta }$)	0.5
Amount of heterogeneity	(${\tau }^{2}$)	0.000, 0.010, 0.025, 0.050, 0.075, 0.100, 0.250, 0.500, 0.750, 1.000
Number of studies	($k$)	10, 30, 50, 70, 90
Average sample size	($N$)	20, 40, 60, 80, 100
Skewness and kurtosis of the random-effects distribution	($s$)	(0, 0), (− 2, 3.65), (− 1, 0.47), (0, − 0.58), (1, 0.51), (2, 3.74)
Replications		1,000

Simulation factors included in the present study, the mathematical nomenclature used in the article, and the values set for each one in the present work

### Analytic procedures

The simulation was programmed in R 4.1.0 [47] using several packages: *PearsonDS* [52] (v1.2.2), *metafor *[54] (v3.1.18), *bootstrap *[55] (v2019.6), *rsample *[56] (v0.1.0), *bayesmeta *[57] (v2.6), *rjags *[58] (v4.10), *R2jags *[59] (v0.6.1), and *runjags *[60] (v2.2.0.2). Complete data and R code files used for the simulation and analyses reported below are available in the supplementary material [32].

In each meta-analysis, all the estimators for the heterogeneity parameter described in the *Background* section were computed. That is, twelve estimators based on the method of moments, two based on maximum likelihood, another two based on least squares, three more focused on artifact correction, two nonparametric estimators, and four Bayesian procedures.

However, the fully Bayesian procedure allows any a priori probability distribution for the parameters of the random-effects model (that is, for ${\mu }_{\delta }$ and ${\tau }^{2}$, since the within-study variances are assumed to be known). To be consistent with previous work that had studied the performance of heterogeneity estimators, we chose seven different combinations of vague or weakly informative *prior*distributions for both parameters following Boedeker & Henson [30]. Specifically, different priors were used to model a function of heterogeneity, whereas for ${\mu }_{\delta }$ the *prior* was a normal distribution with mean and variance equal to 0 and 1000, respectively, in all cases. *Priors* for $\tau$ included two uniform distributions with limits of 0 to 2 and 0 to 100, and three half-Cauchy distributions with scale parameters 1, 5, and 25. Whereas *priors* for precision $(1/{\tau }^{2})$ were $\Gamma (0.001, 0.001)$ and $\Gamma (0.1, 0.1)$.

We would like to point out that previous studies, as discussed by Röver et al., [61] advise against using the inverse gamma distribution to model the variance *prior* because this can force the variance estimate to be positive and often too much probability is allocated to very large heterogeneity values. Additionally, when the ${\mu }_{\delta }$ *prior* is a normal distribution (as set in the present simulation work), the variance of the ${\mu }_{\delta }$ posterior distribution increases proportionally to $\tau$, which can impact the prediction intervals of ${\mu }_{\delta }$, for example. In contrast, half-Cauchy distributions, since they exhibit approximately uniform behavior near zero heterogeneity and monotonically decreasing probability with increasing values of heterogeneity (which guarantees integrability of the lower and upper tails), and bounded uniform distributions, provided that the boundary $[0, a]$is reasonably large, are advisable [61].

Finally, in addition to using seven *prior* distributions, since the posterior distribution for ${\tau }^{2}$ is most likely not symmetric, we chose the posterior mean, median, and mode as the resulting point estimates for the heterogeneity parameter. Therefore, 21 different fully Bayesian procedures for ${\tau }^{2}$ were included in the present simulation work. Overall, we compared the performance of 45 (21 frequentist and 24 Bayesian) point estimators for the heterogeneity parameter.

### Performance criteria

To compare the performance of the heterogeneity estimators described above, we focused on the following outcome variables. First, point estimators were compared in terms of absolute bias, defined as the average difference between the point estimate ${\widehat{\tau }}_{pj}^{2}$ for procedure $p$ and the actual value of ${\tau }^{2}$ along the 1,000 meta-analyses in each simulation condition ($j=1,\dots ,\mathrm{1,000}$),

$$Bias\left({\widehat{\tau }}_{p}^{2}\right)=\frac{{\sum }_{j}\left({\widehat{\tau }}_{pj}^{2}-{\tau }^{2}\right)}{\mathrm{1,000}}$$

Secondly, the precision of the estimates produced by the point estimators is commonly assessed through the *MSE*, which is defined as the variability of the point estimates for procedure $p$ with respect to the actual ${\tau }^{2}$ value across the 1,000 meta-analyses of each condition,

$$MSE\left({\widehat{\tau }}_{p}^{2}\right)=\frac{{\sum }_{j}{\left({\widehat{\tau }}_{pj}^{2}-{\tau }^{2}\right)}^{2}}{\mathrm{1,000}}$$

However, $MSE$ can be decomposed according to the variance and bias of procedure $p$ into $MSE\left({\widehat{\tau }}_{p}^{2}\right)=Var\left({\widehat{\tau }}_{p}^{2}\right)+{\left[Bias\left({\widehat{\tau }}_{p}^{2}\right)\right]}^{2}$, and therefore results regarding $MSE$ may be hiding the variability of the point estimates in those scenarios where procedure $p$ shows a greater bias. This is the reason why the second outcome variable to compare the performance of the heterogeneity estimators was their variance. The variance of estimator $p$ can be defined as the mean squared difference between the point estimate for procedure $p$ and the expected value of these point estimates $E\left[{\widehat{\tau }}_{pj}^{2}\right]$ along the 1,000 meta-analyses in each simulation condition,

$$Var\left({\widehat{\tau }}_{p}^{2}\right)=\frac{{\sum }_{j}{\left({\widehat{\tau }}_{pj}^{2}-E\left[{\widehat{\tau }}_{pj}^{2}\right]\right)}^{2}}{\mathrm{1,000}}$$

Although the variance was our second outcome variable, results regarding the *MSE* were also computed and discussed.

Most of the heterogeneity estimators included in this work are computed analytically and, therefore, a resulting estimate is always guaranteed. Indeed, some of the iterative procedures (i.e., the *r* and *m* estimators) [17] were programmed in such a way that they must always provide an estimate. However, the rest of the iterative estimators (MP, ML, REML, BM, and all fully Bayesian procedures) can lead to convergence problems. Thus, to ensure that 1,000 estimates for each estimation procedure were available across all the simulation conditions, the data of those meta-analyses for which at least one heterogeneity estimator could not be computed were deleted and new data was generated instead. Furthermore, when this occurred, these situations were taken into account, resulting in a non-convergence rate for each iterative procedure in each simulation condition. However, the non-convergence rates of these procedures were equal to zero for all the simulation conditions, except for the ML procedure in only two conditions. The first of these two conditions implied a ${\tau }^{2}=0.75$, $k=10$, $N=20$, and a random-effects distribution with skewness and kurtosis values equal to 1 and 0.51, respectively, while the second simulation condition implied a ${\tau }^{2}=1$, $k=10$, $N=20$, and a random-effects distribution with skewness and kurtosis values equal to 2 and 3.74, respectively. In both conditions, the ML procedure did not converge on only one of the 1,000 meta-analyses generated and, therefore, it was only necessary to generate new data for a single meta-analysis.

## Results

### Absolute bias in normal scenarios

**Fig. 2:**
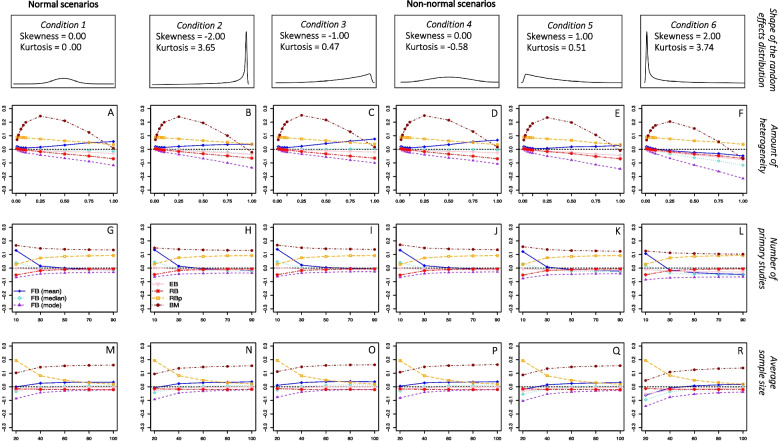
*Absolute bias of the Bayesian estimators* *Note*. Absolute bias of the Bayesian estimators as a function of the amount of heterogeneity, the number of primary studies, and the average sample size. The results are presented separately for each condition of the shape of the random-effects distribution. FB (mean) = fully Bayesian estimators based on the posterior mean; FB (median) = fully Bayesian estimators based on the posterior median; FB (mode) = fully Bayesian estimators based on the posterior mode; RB = Rukhin Bayes estimator; RBp = positive Rukhin Bayes estimator; BM = Bayes Modal estimator

Figures 1 and 2 present the absolute bias of the frequentist and Bayesian estimators, respectively, as a function of the amount of heterogeneity, the number of primary studies, and the average sample size. In addition, these results are presented separately for each condition of the shape of the random-effects distribution. Due to the huge number of estimators available for the heterogeneity parameter, those estimators that showed similar performance were grouped together and their data were averaged to facilitate the understanding of the results. To make the comparison between the frequentist and Bayesian estimators easier, the plots depicted in Figs. 1 and 2 present the same amount of absolute bias (0.60 points) on the y-axis.

Given the main focus of the present work is to compare the performance of these estimators from normal to non-normal random-effects scenarios, we will start by describing the results under normal conditions. The amount of heterogeneity was the simulation factor that affected the bias of the estimators to a greater extent. As can be seen in plot A of Figs. 1 and 2, most estimators obtained slightly positively biased estimates for very low values of ${\tau }^{2}$ (0.01 – 0.025) and more negatively biased estimates as the actual value of ${\tau }^{2}$ increased. However, some estimators showed a different trend. The RBp estimator yielded positively biased estimates regardless of the amount of ${\tau }^{2}$, but followed the general trend of obtaining lower estimates as ${\tau }^{2}$ increased, that is, its positive bias decreased as ${\tau }^{2}$ increased. The BM estimator always overestimated ${\tau }^{2}$ and its bias showed an inverted-U relationship with respect to the amount of heterogeneity, gradually increasing to reach a maximum average absolute bias of 0.23 when the actual ${\tau }^{2}$ value was 0.25 (implying a maximum relative bias of almost 100%). Finally, those fully Bayesian estimators focused on the posterior mean overestimated ${\tau }^{2}$ increasingly the larger the true value of the parameter.

Graphs G and M in Fig. 1 show that the number of primary studies had no noticeable effect on the bias of the frequentist estimators, whereas a large sample size of primary studies reduced the absolute bias: once an average sample size of between 40 and 60 observations was reached, the bias of the frequentist estimators tended to stabilize. The absolute bias of Bayesian estimators stabilized as the number of primary studies increased up to 50 and the average sample size exceeded 60 observations, as presented in plots G and M of Fig. 2.

### Absolute bias in non-normal scenarios

Compared to the normal scenarios, the departure from normality in the distribution of random effects accentuated the effect of (seems to interact with) the amount of heterogeneity for the vast majority of estimators but did not alter the relative ranking of the estimators with regards to their bias.

On the one hand, the order of the heterogeneity estimators with respect to their absolute bias was not altered by the lack of normality, except for the LCHr and LCHm estimators. These estimators showed a medium bias when the normality of the random effects was held (plot A, Fig. 1), whereas in those scenarios where the deviation from normality was most pronounced (plots B and F), these estimators were among the most biased.

On the other hand, the bias trends of most estimators with respect to the amount of heterogeneity became more pronounced as the distribution of random effects departed from normality. That is, in general, even lower estimates were obtained for larger amounts of ${\tau }^{2}$ as the departure from normality increased. This can be seen in the scenarios depicted in plots B and F of Figs. 1 and 2, where the absolute bias of most estimators showed larger negative slopes than in the normal scenario depicted in plot A.

The number of the primary studies did not seem to be a protective factor against a lack of normality in the random effects for the frequentist estimators, but the sample size was shown to attenuate the effect of non-normality on the relationship between the amount of heterogeneity and the bias of some frequentist estimators. Figures S1 and S2 of the supplementary material[32] present a more detailed analysis of the absolute bias of the frequentists estimators as a function of the number and average sample size of primary studies, respectively, the shape of the random-effects distribution. As can be seen, the effect of the lack of normality on the bias of most estimators was very similar regardless of the number of primary studies included in the meta-analysis. However, some heterogeneity estimators (MBH, SJ(CA), MPM, SJ, MP, CA2, DL2, DLm, HSss, ML, and REML) showed similar amounts of bias for meta-analyses of studies with a smaller average sample size in normal scenarios and studies with a larger average sample size in those scenarios where the deviation from normality was most extreme. Although the deviation from normality also affected the bias of these estimators to some extent regardless of the average sample size, its impact was smaller as the sample size increased. It is important to note that, although the CA estimator is represented in the same group as the MBH, SJ(CA) and MPM estimators in Figure S2, the average sample size did not represent a protective factor against non-normality for this first estimator, which remained practically unbiased under both normal and non-normal random-effects scenarios.

The number and average sample size of primary studies also decreased the effect of non-normality on the relationship between the amount of heterogeneity and the bias of most Bayesian heterogeneity estimators. Figures S3 and S4 of the supplemental material [32] present in more detail the bias of the Bayesian estimators as a function of the number and average sample size of primary studies, respectively, and the shape of the random-effects distribution. As can be appreciated, Bayesian estimators showed lower amounts of bias for meta-analyses with 30 studies in non-normal scenarios than for meta-analyses with 10 studies in normal scenarios. Likewise, most of these estimators showed similar amounts of bias for meta-analyses with studies of an average sample size of 40 observations in scenarios where the deviation from normality was most pronounced and for meta-analyses with studies of an average sample size of 20 in normal conditions. Again, although the deviation from normality increased the bias of Bayesian estimators to some extent regardless of the number and average sample size of primary studies, its impact was smaller as the number of primary studies increased.

Finally, along all the simulated scenarios, CA, MBH, SJ(CA), and MPM showed the least biased estimates among the frequentist estimators. Within this group performance was very homogeneous in normal scenarios (the average bias ranged from -0.005 to 0.002), but as the distribution of random effects departed from normality, CA remained essentially unbiased (average bias of 0.003 for the 6^th^ shape of the random-effects distribution) while the MBH, SJ(CA) and MPM estimators slightly over or underestimated ${\tau }^{2}$ depending on the simulated scenario (average bias of 0.006, -0.015 and -0.013, respectively). Furthermore, the bias of these estimators together with SJ, MP, CA2, DL2, DLm, HSss, ML and REML was less influenced by the lack of normality in the random-effects distribution than the rest (LCHr, LCHm, GENQM, DLp, DL, HSk, DLb, HSiv, and HM) of the frequentist estimators.

Concerning Bayesian estimators, those focused on the posterior median showed the lowest bias across all the simulated conditions, followed by the RB estimator. In normal scenarios, the average bias of the fully Bayesian median-centered estimators ranged from -0.021 to 0.005, while the average bias of the RB estimator was 0.019. However, when the distribution of random effects departed from normality, the absolute bias increased for these Bayesian procedures (the average bias ranged from -0.052 to -0.024 for the fully Bayesian median-centered estimators in the 6^th^ shape of the random-effects distribution), while became negative for the RB estimator (average bias of -0.018). In addition, it is worth noting that the absolute bias of the RB estimator (together with that of RBp) was less affected by non-normality than that of the fully Bayesian and the BM estimators.

### Variance in normal scenarios

**Fig. 3:**
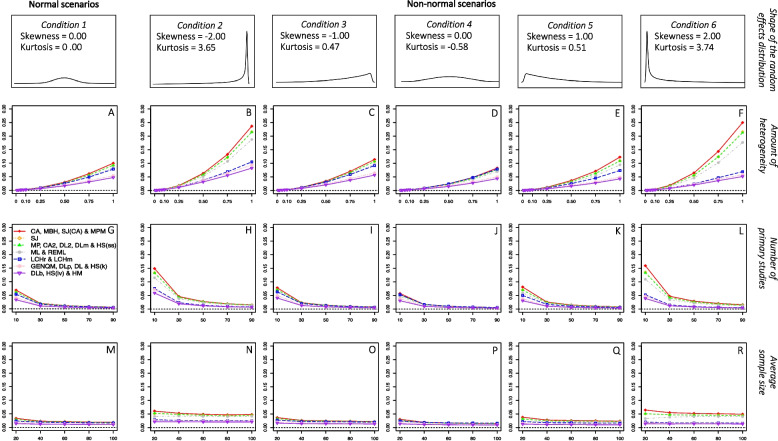
*Variance of the frequentist estimators* *Note*. Variance of the frequentist estimators as a function of the amount of heterogeneity, the number of primary studies, and the average sample size. The results are presented separately for each condition of the shape of the random-effects distribution. CA = Cochran estimator; MBH = Malzahn-Böhning-Holling estimator; SJ(CA) = Sidik-Jonkman estimator with prior CA estimation; MPM = median-unbiased Mandel-Paule estimator; SJ = Sidik-Jonkman estimator; MP = Mandel-Paule estimator; CA2 = two-step Cochran estimator; DL2 = two-step DerSimonian-Laird estimator; DLm = multistep DerSimonian-Laird estimator; HS(ss) = Hunter-Schmidt estimator weighted by sample size; ML = maximum likelihood estimator; REML = restricted maximum likelihood estimator; LCHr = Lin-Chu-Hodges *r* estimator; LCHm = Lin-Chu-Hodges *m* estimator; GENQM = median-unbiased generalized *Q* statistic estimator; DLp = positive DerSimonian-Laird estimator; DL = DerSimonian-Laird estimator; HS(k) = Hunter-Schmidt estimator corrected by small sample size; DLb = nonparametric bootstrap DerSimonian-Laird estimator; HS(iv) = Hunter-Schmidt estimator weighted by inversed variance; HM = Hartung-Makambi estimator

**Fig. 4:**
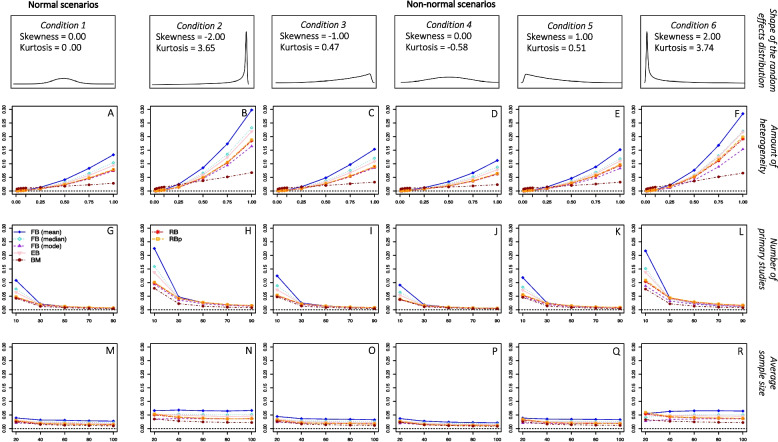
*Variance of the Bayesian estimators* *Note*. Variance of the Bayesian estimators as a function of the amount of heterogeneity, the number of primary studies, and the average sample size. The results are presented separately for each condition of the shape of the random-effects distribution. FB (mean) = fully Bayesian estimators based on the posterior mean; FB (median) = fully Bayesian estimators based on the posterior median; FB (mode) = fully Bayesian estimators based on the posterior mode; RB = Rukhin Bayes estimator; RBp = positive Rukhin Bayes estimator; BM = Bayes Modal estimator

Figures 3 and 4 present the variance of the frequentist and Bayesian estimators, respectively, as a function of the amount of heterogeneity, the number of primary studies, and the average sample size. These results are presented separately for each condition of the shape of the random-effects distribution. To make the comparison between the frequentist and Bayesian estimators easier, the plots depicted in Figs. 3 and 4 present the same amount of variance (0.30 points) on the y-axis.

Again, since the main goal of the present work is to compare the performance of these estimators from normal to non-normal random-effects scenarios, we will start by describing the results under normal conditions. In general terms, the variance of all heterogeneity estimators decreased as the true amount of heterogeneity became smaller and as the number of the primary studies became larger. Although the efficiency of all heterogeneity estimators increased slightly as the average sample size became larger, this simulation factor did not affect the variance of the heterogeneity estimators to the same extent as the amount of heterogeneity and the number of studies.

Considering the amount of heterogeneity, it can be seen in plot A of Figs. 3 and 4 that all estimators showed greater variance as the heterogeneity increased, except for the BM estimator which showed a different trend. Although the variance of the BM estimator also increased as ${\tau }^{2}$ increased, it was more variable than the rest of estimators for ${\tau }^{2}$ values between 0.01 and 0.25 but obtained the lowest variances when ${\tau }^{2}$ exceeded 0.50.

### Variance in non-normal scenarios

Deviation from normality in the distribution of random effects accentuated the effect of (seemed to interact with) the amount of heterogeneity and the number of studies for most estimators. That is, in general, greater variances were obtained for larger amounts of ${\tau }^{2}$ as the departure from normality increased, as can be seen in the scenarios depicted in plots B and F of Figs. 3 and 4, where the variance of most estimators showed larger positive slopes compared to the normal scenario depicted in plot A. Furthermore, even larger variances were also obtained for a smaller number of studies as the departure from normality was more extreme, as shown in plots H and L of Figs. 3 and 4 compared to the normal scenario depicted in plot G. However, the order of the estimators with respect to their variance (variance ratio) did not alter from normal to non-normal scenarios.

The number of primary studies was shown to decrease the effect of non-normality on the relationship between the amount of heterogeneity and the variance of all heterogeneity estimators. Figures S5 and S6 of the supplemental material [32] present a more detailed analysis of the variance of the frequentists and Bayesian estimators, respectively, as a function of the number of primary studies and the shape of the random-effects distribution. As can be seen, most heterogeneity estimators showed similar amounts of variance for meta-analyses with 10 studies in normal scenarios and for meta-analyses with 30 studies in those scenarios where the deviation from normality was most extreme. Although the deviation from normality affected the variance to some extent regardless of the number of primary studies, its impact was smaller as the number of primary studies increased. On the contrary, the average sample size of the primary studies did not seem to be a protective factor against a lack of normality in the random effects, since the slopes of the lines for the variance of the different estimators in the condition where normality was held were not altered in those scenarios where the deviation from normality was greater. This can be also seen in Figures S7 and S8 of the supplemental material, which present in more detail the variance of the frequentists and Bayesian estimators, respectively, as a function of the average sample size of primary studies and the shape of the random-effects distribution.

Finally, those estimators that had previously shown to be less biased, showed greater variances than the rest of the estimators analyzed. Among the frequentist estimators, CA, MBH, SJ(CA), and MPM were the most variable both, in normal (the average variance ranged from 0.022 to 0.024) and non-normal scenarios (the average variance ranged from 0.048 to 0.061 for the 6^th^ shape of the random-effects distribution). The frequentist estimators that showed to be most efficient were the DLb, HSiv and HM estimators both, in normal (the average variance ranged from 0.011 to 0.012) and non-normal conditions (the average variance ranged from 0.013 to 0.014 for the 6^th^ shape of the random-effects distribution).

Within the Bayesian procedures, those fully Bayesian estimators centered on the posterior median and the RB estimator yielded intermediate variances among the Bayesian estimators analyzed both, in normal (the average variance ranged from 0.018 to 0.028) and non-normal scenarios (the average variance ranged from 0.041 to 0.056 for the 6^th^ shape of the random-effects distribution). The Bayesian estimator that showed to be the most efficient was the BM estimator, obtaining an average variance of 0.014 in normal scenarios, and 0.026 for the 6^th^ shape of the random-effects distribution.

To conclude the *Results* section, we briefly present how the bias and variance results translate into *MSE*. Supplemental materials [32] also include Figures S9 and S10 that present the *MSE* of the frequentist and Bayesian estimators, respectively, as a function of the amount of heterogeneity, the number of primary studies, and the average sample size. These results are also presented separately for each condition of the shape of the random-effects distribution. To make the comparison between the frequentist and Bayesian estimators easier, the plots showing *MSE* depicted in Figures S9 and S10 have the same y-axis (0–0.35 points).

In normal random-effects scenarios, all the heterogeneity estimators obtained similar results regarding *MSE*. In these conditions, the simulation factor that most affected the *MSE* was the amount of heterogeneity: Most estimators showed a greater *MSE* as the amount of heterogeneity increased (except for the BM estimator). At the same time, most estimators showed an increase in their *MSE* for those scenarios in which meta-analyses with fewer than 30 studies and an average sample size of less than 40 observations were simulated.

Although all the heterogeneity estimators obtained similar results regarding *MSE*, the deviation from normality resulted in an increase of the *MSE* of all the estimators and accentuated the differences in terms of *MSE* among the estimators analyzed.

## Discussion

The present paper is focused on the performance of the point heterogeneity estimators under conditions where the distribution of random effects departs from normality compared to normal scenarios. For this purpose, we carried out a Monte Carlo simulation study where data for meta-analyses based on the standardized mean difference were generated. In total, 21 frequentist and 24 Bayesian estimators have been compared in terms of absolute bias and variance, including several procedures (LCHm, LCHr, MPM, and GENQM) [17, 19] that have not been compared in similar simulation studies so far.

One of our main goals was to answer the question of whether the best estimator under normal parametric conditions remained the same in non-normal conditions. In this respect, our results show that, fortunately, the estimators ranking in terms of their absolute bias and variance does not change when the normality in the distribution of parametric effects is altered (except for the LCHr and LCHm estimators). We consider that these are good news for meta-analysts since our results suggest that it is not needed to choose one estimator or another depending on how the random effects are believed to be distributed.

However, the conclusion cannot be that a lack of normality in the distribution of parametric effects has no implications for the estimation of ${\tau }^{2}$. Although the magnitude of its effect may differ from one procedure to another, most estimators obtained lower mean ${\tau }^{2}$ estimates and greater variances as the parametric distribution deviated from normality. Moreover, since some estimators showed to be more influenced by the non-normality than others, the variability among the mean ${\tau }^{2}$ estimates increased as the distribution of random effects deviates from normality. Our results in this regard may partly explain why the ${\tau }^{2}$estimates computed for the study of Shadish and Baldwin [41], which summarized estimates whose distribution was farther from normal, vary to a greater extent (from 0.004 to 1.18) than for the study of Richards and Richardson [40] (${\widehat{\tau }}^{2}$ range goes from 0.11 to 0.39).

In general, we could say that those estimators free from normality assumptions are not necessarily the most robust when this assumption does not meet. In fact, among the frequentist procedures based on Cochran's $Q$statistic that do not necessarily assume any assumption of normality, some of them have shown less bias (and/or variance) and others more than the procedures based on maximum likelihood or weighted least squares, which do assume this assumption. On the other hand, the estimators developed by Rukhin [27], which are more flexible but assume certain assumptions derived from the normality of the random effects, hardly suffer any variation in their performance in non-normality scenarios where the kurtosis have been altered.

Our results suggest that a large average sample size of primary studies seems to be a protective factor against non-normality with respect to the bias of most Bayesian, but only of some frequentist heterogeneity estimators. That is, lower estimates of ${\tau }^{2}$ were obtained the less normal the distribution of random effects was. Nevertheless, most of the Bayesian and some frequentist estimators analyzed (MBH, SJ(CA), MPM, SJ, MP, CA2, DL2, DLm, HSss, ML, and REML) showed similar amounts of bias for smaller average sample sizes in normal scenarios and for larger average sample size in those scenarios where the deviation from normality was most extreme. Indeed, this same trend was found for Bayesian estimators with respect to the number of primary studies included in the meta-analysis. With respect to the variance of the estimators analyzed, the number of studies (but not their average sample size) seems to be a protection factor against a lack of normality. In other words, most heterogeneity estimators showed similar amounts of variance for meta-analyses with 10 studies in normal scenarios and for meta-analyses with 30 studies in those scenarios where the deviation from normality was most extreme. Indeed, the effect of non-normality became smaller as the number of primary studies increased.

Regarding the new estimators tested, the procedures proposed by Lin et al. [17] exhibited substantial bias in normal scenarios, which increase as the distribution of random effects departed from normality. However, although they are not among the most efficient estimators, the variability of their estimates was not altered by the non-normality of the random effects. Concerning the estimators proposed by Viechtbauer [19], the MPM estimator was on average slightly less biased than the MP estimator across the simulated scenarios, but also slightly more variable. Specifically, under less favorable conditions, the estimated bias of MPM was 33% lower than that of MP whereas the variability of the former was 15% higher than that of the latter. The GENQM estimator showed to be substantially more biased than MPM, nevertheless, this difference in bias became practically null the more bias both estimators showed, and as the random-effects distribution deviated from normality. In contrast, the variance of the GENQM estimates was between 40 and 75% lower than that of MPM.

With respect to previous findings, our results agree with those of Kromrey and Hogarty [42] in that, in general terms, the CA estimator appears to be less biased than DL and ML. However, according to their results, CA showed more bias than DL and ML in non-normal settings for primary studies with small sample sizes. This last finding differs from the results found in our work, where the bias of CA was minimally affected by the sample size of the primary studies, and in no case showed a bias greater than that of ML or DL. In addition, we have not found the sample size to be a protective factor against non-normality as Kromrey and Hogarty [42] seem to suggest with respect to the CA estimator. These authors stated that CA was essentially unbiased in normal settings, reaching a maximum bias of 0.07 with samples averaging 10 subjects per primary study (regardless of the number of primary studies). While in non-normal scenarios, this bias reached 0.69 for an average sample size of 10 and decreased to 0.03 with sample sizes of 200 observations. The difference in the slope of the lines that would model the decrease in the CA bias as a function of the average sample size would have led us to think that this factor may have a protective role against non-normality: obtaining similar amounts of bias between normal and non-normal scenarios with relatively large samples. However, we have not found such a difference in our results.

One possible reason to explain this inconsistency could be the fact that the sample sizes that these authors simulated were twice as small and large as ours (ranged from an average of 10 to 200 observations per primary study, whereas ours ranged from an average of 20 and 100). To find out whether this was the underlying explanation for the differences between our results, we carried out a small simulation in which the conditions mentioned by Kromrey and Hogarty [42] in their results were replicated. Figure S11, available at the supplementary material [32], shows the results obtained. Once more, these results did not show that the CA estimator was more biased than the DL and ML estimators under non-normal conditions, even for very small average sample sizes such as 10 observations per primary study. The bias of CA also did not decrease when the average sample size increased. Nonetheless, we found that the bias of the DL procedure decreased and stabilized around -0.4 when changing from an average sample size of 10 to 20 observations per study, something we could not observe previously since sample sizes below 20 were not included in our simulation study.

Our results for those conditions where the normality of the random effects distribution was held ($$s=1)$$ agree in many respects with the findings of those previous simulation studies [21, 30, 43–45] comparing different heterogeneity estimators under normal random-effects scenarios. Although Viechtbauer [21] used average sample sizes six times larger than those used in our study (40, 80, 160, 320 and 640) and $${\tau }^{2}$$ values ten times smaller (0, 0.01, 0.025, 0.05 and 0.1), our study replicated the results found in this previous simulation in terms of *MSE*, efficiency, and bias, with the exception that Viechtbauer [21] found the CA estimator to be unbiased in all conditions, whereas in our study it presented a slight positive bias (maximum 0.007) that decreased as $${\tau }^{2}$$ reached 0.1. Likewise, our results also suggest that DL and REML present a similar bias for the range of $${\tau }^{2}$$values studied in Viechtbauer [21]. However, our study evaluates scenarios where $${\tau }^{2}$$ reaches a maximum value of 1 and we found that REML and ML have a smaller and more similar bias as $${\tau }^{2}$$ increases (maximum bias around -0.1) than DL and HSiv (around -0.2).

Although the ${\tau }^{2}$ values examined in the work of Novianti et al. [43] are extremely small (ranged from 0 to 0.0366) compared to those examined in our simulation, we agree with these authors in that, when the heterogeneity ranges from 0 to 0.05, the bias of all estimators analyzed by that these authors is relatively small and comparable, except for the SJ procedure which greatly overestimates the heterogeneity in all cases. At the same time, our results show, like those of Novianti et al. [43] that, as heterogeneity increases, the absolute bias of the analyzed procedures increases while the relative bias decreases. On the other hand, Novianti et al. [43] do not directly provide results referring to the efficiency of the estimators, but the trends they suggest (i.e., that efficiency decreases as ${\tau }^{2}$ increases and $k$ and $N$ decrease) are compatible with our findings.

Perhaps, the previous simulation study with which our results are least consistent is that of Petropoulou and Mavridis [44], who only reported results regarding the absolute bias of the estimators analyzed. These authors concluded that the bias of all estimators increased as heterogeneity increased, as we found in the present work, and that it decreased as the number of primary studies increased. Our results, on the contrary, show that the bias of the estimators decreases markedly as the sample size increases, but almost negligibly as the number of primary studies increases, although this decreasing trend depends on the procedure evaluated. While Petropoulou and Mavridis [44] claimed that REML was less biased than MP in most scenarios, we found just the opposite except for those scenarios where ${\tau }^{2}$ ranged from 0.01 to 0.025. We also did not find DLp and DLb to have a small bias in all conditions and to be the best performing procedures, in fact, our results point to them being among the most biased frequentist estimators as heterogeneity increases. Nor do we find the HM estimator to be the least biased when the heterogeneity ranges from 0.01 to 0.05, in fact it is one of the most biased frequentist estimators in these scenarios. Finally, we agree with Petropoulou and Mavridis [44] in that the RBp, SJ and BM procedures have a non-negligible positive bias for a very wide range of heterogeneity values and should be avoided.

Our results agree with those found by Langan et al. [45] for the most part, except for the following points. These authors found that the DL procedure was one of the least negatively biased estimators, distinctly lower than MP for a medium number of studies. However, we found that MP always obtains a lower or similar bias to DL. While Langan et al.[45] claimed that HM has a comparatively lower *MSE* than the rest of the estimators in all scenarios, our results show that this procedure has one of the highest *MSE* among the frequentist estimators considered. To conclude, these authors also concluded that SJ(CA) showed a larger and positive bias as the sample size of the studies increased, whereas we found the opposite trend.

The simulation conditions of the study of Boedeker and Henson [30] are the most similar to those of the present work. This potentially explains why our results agree for the most part, except for the *prior* specification recommended for modeling the heterogeneity parameter in the fully Bayesian procedure. Our results agree with those of Boedeker and Henson [30] in that those fully Bayesian estimators centered on the posterior median were less biased than those centered on the posterior mean and mode, which in comparison tended to obtain larger and smaller ${\tau }^{2}$ estimates, respectively. However, we found that the procedure based on $\left(\frac{1}{{\tau }^{2}}\right)\sim \Gamma (\mathrm{0.001,0.001})$ *prior* specification obtains estimates with a noticeably larger negative bias as heterogeneity increases than other *prior* distributions for $\tau$, such as $U(\mathrm{0,100})$ and $U(\mathrm{0,2})$, or half-Cauchy distributions with scale parameters 5 and 25.

### Practical recommendations

The implications of an improper estimation of the heterogeneity parameter due to the non-normality of the random-effects distribution are diverse: While the mean effect and its confidence interval have been shown to be relatively robust against non-normal conditions [1–3], its influence on the estimation of prediction intervals appears to be important [62, 63]. Returning to the two meta-analyses we used as examples in the *Background* section, for the study of Richards and Richardson [40], depending on the procedure chosen for estimating the heterogeneity parameter, prediction intervals computed from the 45 ${\tau }^{2}$ estimators here analyzed range from [-1.24, 0.12] to [-1.832, 0.6633], the width of the latter being 1.84 times the width of the former. This is not the case for the Shadish and Baldwin [41] study, where the distribution of effect estimates was more deviated from normality than in the previous case: prediction intervals range from [0.42, 0.77] to [-1.36, 2.99], the latter being more than twelve times wider than the former.

To be aware of the potential threat of non-normal random effects to the results of our meta-analysis, statistical tests have been developed to assess the possible deviation from normality [64]. However, the statistical power of these procedures may be inadequate. Another tentative solution, if we suspect that the distribution of parametric effects may not follow a normal distribution, is the application of more flexible models [62]. Nevertheless, the use of these models could result in an overfitting of the data if they are not applied correctly and, at the moment, the lack of these models in everyday software makes their implementation difficult.

In addition to the above, heterogeneity parameter estimators often exhibit a feature that makes it even more difficult to choose the best one: an inverse relationship between bias and efficiency, as evidenced also by previous works [21, 30, 43–45]. In other words, those estimators that tend to show less biased estimates are also those that tend to show more variability in their estimates, whereas none of the estimators actually dominates the others in terms of *MSE*. For example, the MP and REML are among the most advised frequentist estimators [9, 21, 30, 43–45]. For conditions simulated with skewness = 2 and kurtosis = 3.74, the estimated MP bias reached as high as -0.14 with ${\tau }^{2}=1$, $k=10$, and $N=20$, whereas that of REML reached as high as -0.27. However, for the same conditions the estimated variances of the MP and REML estimator were 0.65 and 0.48, respectively.

Despite the difficulties encountered, our results indicate that there are several estimators less biased that, although they show higher sample variances than the rest, in meta-analyses with a minimum number and average sample size of the primary studies seem to be the best option. Within the frequentist framework, CA, MBH, SJ(CA), and MPM, followed by MP, CA2, DL2, DLm, and HSss, showed the least bias although highest variance in most conditions. However, in meta-analyses with at least 90 studies with an average sample size of 60 observations, these estimators obtain a reasonably maximum amount of bias as the rest of the frequentist estimators given their decrease in sample variability, even in non-normal random-effects scenarios. If a procedure based on maximum likelihood is preferred, REML showed to be less biased but more variable than ML, although it obtained a higher bias than the estimators mentioned above for most of the simulated conditions. For REML to obtain a similar maximum bias as the rest of the estimators, also in non-normal conditions, the average sample size should increase to 100 observations per primary study. In many actual meta-analyses these conditions will not be easily met, and in these cases our recommendation for applied meta-analysts is to evaluate the range of ${\widehat{\tau} }^{2}$ values that can be obtained from their data, as well as the implications that this variability may have on the pooled effect, its confidence interval and the prediction interval. To facilitate this task, we have developed the *tau2()* function in the free software R that allow to obtain the range of ${\widehat{\tau} }^{2}$ values obtained from the 45 estimators analyzed in this work from the effect estimates and the sample size of the primary studies. This function also returns the range for the combined effect size, its confidence interval, and its prediction interval, so that meta-analysts have an easy-to-use tool to help them report these results as sensitivity analyses until new statistical methods are developed that do not present these difficulties. The code of the *tau2()*function as well as an explanatory document are available as part of the supplementary material [32].

From a Bayesian perspective, those fully Bayesian procedures centered on the posterior median with $\tau$ *prior* specifications such as $U(0,100)$ and $U(\mathrm{0,2})$, or half-Cauchy distributions with scale parameters 5 and 25, showed less biased estimates than most of the Bayesian procedures in normal random-effects conditions, including the RB estimator. In spite of this, as the random-effects distribution departed from normality, these fully Bayesian estimators became more negatively biased while RB was insensitive to deviations from normality, and it has also showed a lower variance. In the event that it was necessary to employ a fully Bayesian procedure, we recommend applied meta-analysts to compute both, the posterior median and posterior mean, when their distribution of effect estimates departs substantially from normality. The reason behind is that all fully Bayesian procedures tend to obtain lower ${\tau }^{2}$ estimates as the deviation from normality increase. But, since those centered on the posterior mean always produce greater estimates than those centered on the posterior median, the formers yielded less biased ${\tau }^{2}$ estimates when the deviation from normality became extreme.

### Limitations and future research

Given the huge number of simulation conditions, factors that could have important implications for the results have been omitted. One of these factors is the ${I}^{2}$ index, that is, the percentage of the total variability present in the effect estimates that is due to heterogeneity variance. Another two factors regarding the primary studies are the homogeneity of sizes and variances between the control and experimental groups. The works of Kromrey and Hogarty [42] and Langan et al. [45] lead us to believe that these factors could have implications for the bias and efficiency of ${\tau }^{2}$ point estimators, and we do believe that their effect should be evaluated in future studies. Some of the procedures for creating intervals around ${\tau }^{2}$ do not require previous point estimates, but others do, so including them in the present work would have substantially increase the number of analyzed procedures and would have required a higher amount of time and computational resources, which is why interval estimation of the heterogeneity parameter has not been addressed in the present work either. For those readers interested in providing a range of values for the heterogeneity parameter, we refer them to the simulation studies carried out by Boedeker and Henson [30] and Viechtbauer [65] for those scenarios in which the random effects follow and deviate from a normal distribution, respectively.

Another limitation of this work is that we were not able to explain why previous simulation studies [21, 30, 43–45] reached conclusions that are sometimes contradictory to the results of the present work. It is to be expected that these differences are due to the use of different simulation factors and the different levels set for each of them. However, it would be necessary to examine the possibility that these discrepancies are due to differences in the way the primary data were generated or in the code for computing many of the heterogeneity estimators that were not included in user-friendly software at the time the simulation study was conducted. Therefore, we would like to draw attention to the need to host the simulation code in one of the web repositories that have become so widespread, as well as the simulation data generated from it, since sometimes the lack of appropriate resources makes it difficult to run the code to replicate a simulation work.

Beyond the fact that different factors with varying levels are used in each simulation study, the overall findings reflect the great complexity involved in estimating the heterogeneity parameter in random-effects meta-analyses. It could be thought that an important limitation of this type of studies is the lack of clear guidelines on which estimator to use. However, even knowing how all the factors that can be controlled or evaluated by the meta-analyst (deviation from normality in the distribution of effect estimates, number and average sample size of primary studies, amount of ${I}^{2}$, degree of homoscedasticity and equality of sample sizes between comparison groups, etc.) affect the ${\tau }^{2}$ estimation, we still could not choose which is the best estimator since the amount of actual heterogeneity is the main factor that affects its own estimation and remains unknown.

Finally, we would like to emphasize that our simulation is based on meta-analyses of standardized mean differences and, therefore, the results presented in this article can only be extrapolated to meta-analyses based on this same effect size index or others that are also asymptotically normally distributed.

## Conclusions

The present work highlights the role that the deviation from normality may be playing in the conclusions of the meta-analyses that are carried out on a daily basis. Although the estimation and inference of the combined effect have proven to be sufficiently robust to the non-normality of random effects, the estimation of the heterogeneity parameter appears to be affected to a greater extent. Real studies have been used to show how the estimation of ${\tau }^{2}$ may be impacted and how the conclusions of the prediction interval may vary, depending on the estimator chosen. Also, it should not be overlooked how variations in the estimated amount of heterogeneity may influence the conclusions of subsequent analyses of moderator variables.

We have also taken the opportunity to compare the performance of several new random-effects variance estimators with previous procedures. And, at the same time, we have made available an R function that will allow meta-analysts to obtain out of their data the range of ${\tau }^{2}$ values computed from the 45 estimators analyzed in this work, as well as to assess how the pooled effect, its confidence interval, and its prediction interval vary according to the estimator chosen. The underlying idea is, in words of Kromrey and Hogarty [42]: “[…] to exercise caution in the interpretation of the results obtained from random-effects models” and, also, “highlight the need for the development of meta-analytic methods that are robust to violations of these assumptions”.

## Supplementary Information

**Additional file 1.** Absolute bias of the frequentist estimators as a function of the number of primary studies.

**Additional file 2. **Absolute bias of the frequentist estimators as a function of the average sample size of primary studies*.*

**Additional file 3.** Absolute bias of the Bayesian estimators as a function of the number of primary studies.

**Additional file 4.** Absolute bias of the Bayesian estimators as a function of the average sample size of primary studies.

**Additional file 5.** Variance of the frequentist estimators as a function of the number of primary studies.

**Additional file 6.** Variance of the Bayesian estimators as a function of the number of primary studies.

**Additional file 7.** Variance of the frequentist estimators as a function of the average sample size of primary studies.

**Additional file 8.** Variance of the Bayesian estimators as a function of the average sample size of primary studies.

**Additional file 9. ** Mean squared error of the frequentist estimators.

**Additional file 10.** Mean squared error of the Bayesian estimators.

**Additional file 11.** Absolute bias for the CA, DL and ML estimators according to some simulation conditions set for the study of Kromrey & Hogarty.

## Data Availability

The datasets generated and analyzed during the current study are available in the Open Science Framework repository, https://osf.io/bv4au/?view_only=ec3ac26dad1d45efa9a65dd3cb88cdb5.33

## Abbreviations

CA: Cochran heterogeneity estimator
MP: Mandel-Paule heterogeneity estimator
DL: DerSimonian-Laird heterogeneity estimator
HM: Hartung-Makambi heterogeneity estimator
CA2: Two-step Cochran heterogeneity estimator
DL2: Two-step DerSimonian-Laird heterogeneity estimator
DLp: Positive DerSimonian-Laird heterogeneity estimator
LCHr: Lin-Chu-Hodges *r* heterogeneity estimator
LCHm: Lin-Chu-Hodges *m* heterogeneity estimator
DLm: Multistep DerSimonian-Laird heterogeneity estimator
MPM: median-unbiased Mandel-Paule heterogeneity estimator
GENQM: median-unbiased generalized *Q* heterogeneity estimator
ML: Maximum likelihood heterogeneity estimator
REML: Restricted maximum likelihood heterogeneity estimator
SJ: Sidik-Jonkman heterogeneity estimator
SJ(CA): Sidik-Jonkman heterogeneity estimator based on a prior CA estimation
FB: Fully Bayesian heterogeneity estimators
RB: Rukhin Bayes heterogeneity estimator
RBp: Positive Rukhin Bayes heterogeneity estimator
BM: Bayes Modal heterogeneity estimator
DLb: Non-parametric bootstrap DerSimonian-Laird heterogeneity estimator
MBH: Malzahn-Böhning-Holling heterogeneity estimator
HSiv: Hunter-Schmidt heterogeneity estimator weighted by inversed variance
HSss: Hunter-Schmidt heterogeneity estimator weighted by sample size
HSk: Hunter-Schmidt heterogeneity estimator corrected by small sample size
