# Supplementary material for: Heterogeneity estimation in meta-analysis of standardized mean differences when the distribution of random effects departs from normal: A Monte Carlo simulation study
Source: BMC Med Res Methodol. 2023 Jan 17;23:19. doi: 10.1186/s12874-022-01809-0 (PMC9843903; doi:10.1186/s12874-022-01809-0)
Supplement: Supplementary file 6 — Additional file 6. Variance of the Bayesian estimators as a function of the number of primary studies. [file 12874_2022_1809_MOESM6_ESM.pdf]

**Figure S6**

*Variance of the Bayesian estimators as a function of the number of primary studies*

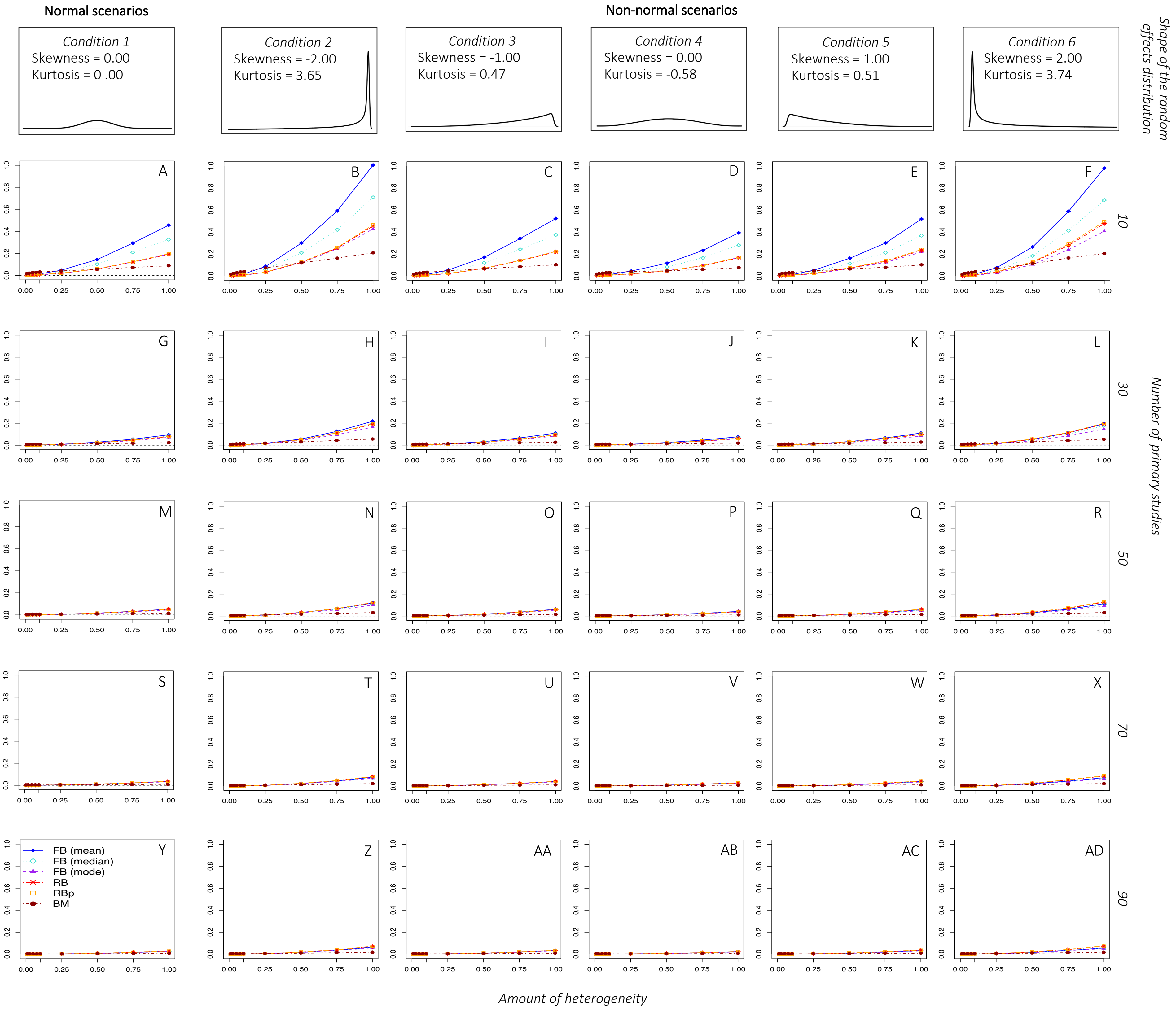


*Note.* Variance of the Bayesian estimators as a function of the number of primary studies, the amount of heterogeneity and the shape of the random-effects distribution. FB (mean) = fully Bayesian estimators based on the posterior mean; FB (median) = fully Bayesian estimators based on the posterior median; FB (mode) = fully Bayesian estimators based on the posterior mode; RB = Rukhin Bayes estimator; RBp = positive Rukhin Bayes estimator; BM = Bayes Modal estimator.
