# Supplementary material for: Heterogeneity estimation in meta-analysis of standardized mean differences when the distribution of random effects departs from normal: A Monte Carlo simulation study
Source: BMC Med Res Methodol. 2023 Jan 17;23:19. doi: 10.1186/s12874-022-01809-0 (PMC9843903; doi:10.1186/s12874-022-01809-0)
Supplement: Supplementary file 7 — Additional file 7. Variance of the frequentist estimators as a function of the average sample size of primary studies. [file 12874_2022_1809_MOESM7_ESM.pdf]

Figure S7

Variance of the frequentist estimators as a function of the average sample size of primary studies

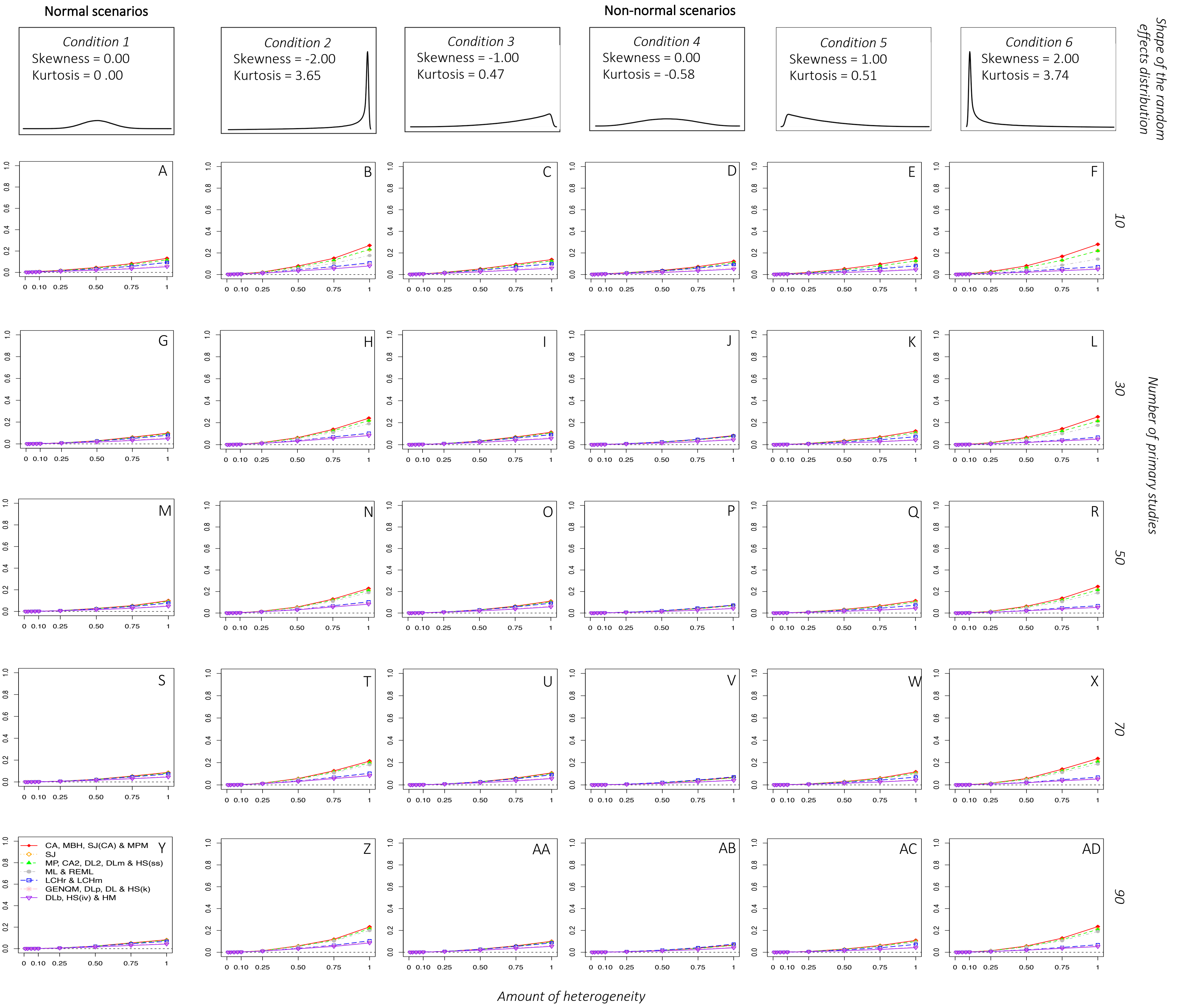

Note. Variance of the frequentist estimators as a function of the average sample size of primary studies, the amount of heterogeneity and the shape of the random-effects distribution. CA = Cochran estimator; MBH = Malzahn-Böhning-Holling estimator; SJ(CA) = Sidik-Jonkman estimator with prior CA estimation; MPM = median-unbiased Mandel-Paule estimator; SJ = Sidik-Jonkman estimator; MP = Mandel-Paule estimator; CA2 = two-step Cochran estimator; DL2 = two-step DerSimonian-Laird estimator; DLm = multistep DerSimonian-Laird estimator; HS(ss) = Hunter-Schmidt estimator weighted by sample size; ML = maximum likelihood estimator; REML = restricted maximum likelihood estimator; LCHr = Lin-Chu-Hodges *r* estimator; LCHm = Lin-Chu-Hodges *m* estimator; GENQM = median-unbiased generalized *Q* statistic estimator; DLp = positive DerSimonian-Laird estimator; DL = DerSimonian-Laird estimator; HS(k) = Hunter-Schmidt estimator corrected by small sample size; DLb = nonparametric bootstrap DerSimonian-Laird estimator; HS(iv) = Hunter-Schmidt estimator weighted by inversed variance; HM = Hartung-Makambi estimator.
