# Supplementary material for: Heterogeneity estimation in meta-analysis of standardized mean differences when the distribution of random effects departs from normal: A Monte Carlo simulation study
Source: BMC Med Res Methodol. 2023 Jan 17;23:19. doi: 10.1186/s12874-022-01809-0 (PMC9843903; doi:10.1186/s12874-022-01809-0)
Supplement: Supplementary file 11 — Additional file 11. Absolute bias for the CA, DL and ML estimators according to some simulation conditions set for the study of Kromrey & Hogarty. [file 12874_2022_1809_MOESM11_ESM.pdf]

Figure S11

Absolute bias for the CA, DL and ML estimators according to some simulation conditions set for the study of Kromrey & Hogarty

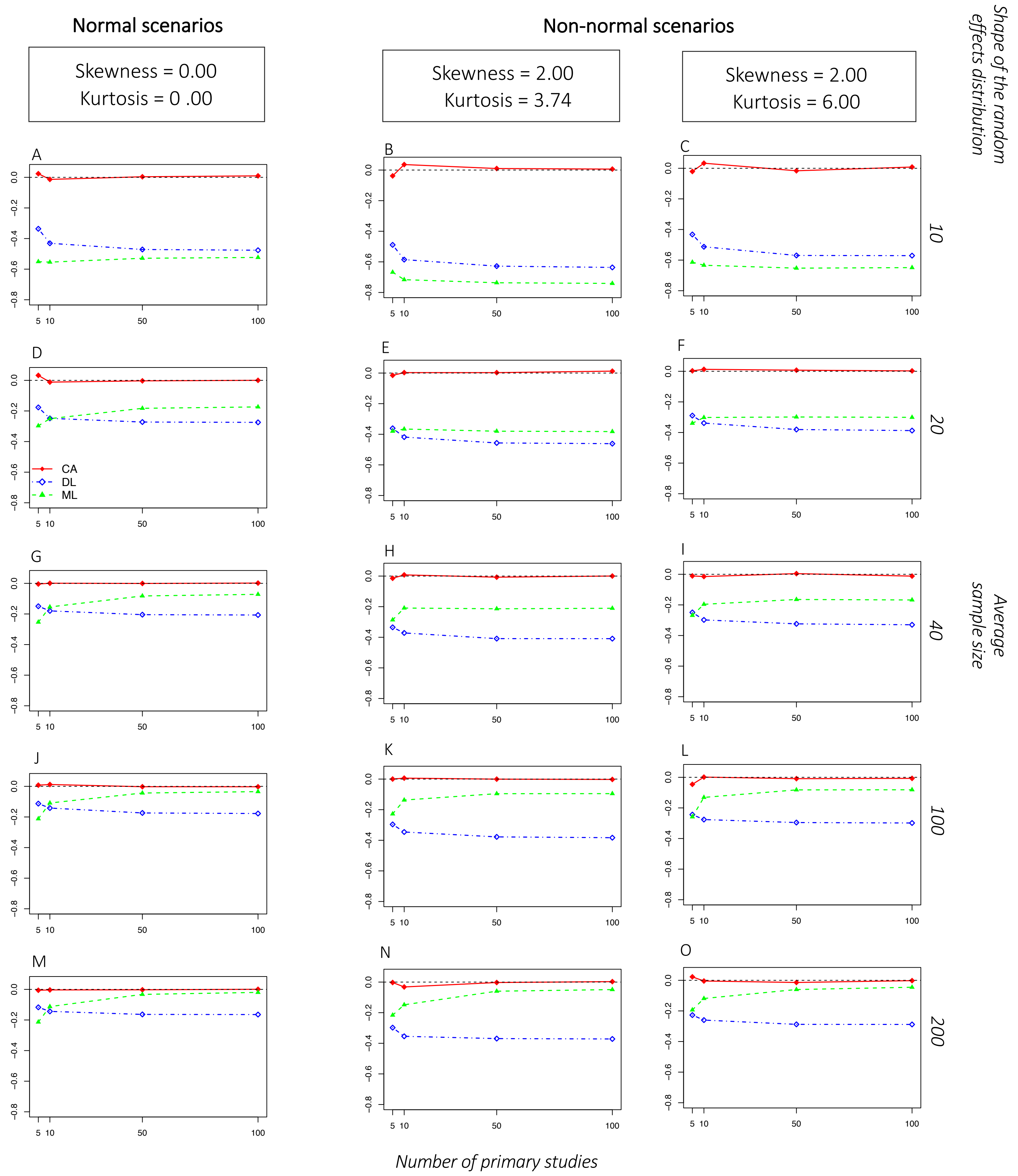

Note. Absolute bias of the CA, DL and ML estimators as a function of the number of primary studies and the average sample size. The results are presented separately for each condition of the shape of the random-effects distribution, where the overall effect (μ_δ) and the between-study variance (τ^2) are set to 0.5 and 1, respectively. CA = Cochran estimator; DL = DerSimonian-Laird estimator; ML = maximum likelihood estimator.
